# A Randomized, Open-Label, Single-Dose, Crossover Study of the Comparative Bioavailability of EPA and DHA in a Novel Liquid Crystalline Nanoparticle-Based Formulation of ω-3 Acid Ethyl Ester Versus Omacor^®^ Soft Capsule among Healthy Adults

**DOI:** 10.3390/ijms242417201

**Published:** 2023-12-06

**Authors:** Kwi-Man Kang, Sang-Won Jeon, Anindita De, Tae-Sun Hong, Young-Joon Park

**Affiliations:** 1 College of Pharmacy, Ajou University, Worldcup-ro 206, Yeongtong-gu, Suwon-si 16499, Republic of Korea; kwiman@ajou.ac.kr (K.-M.K.); aninditanirupa@gmail.com (A.D.); 2 Research Center, IMDpharm Inc., 17, Daehak 4-ro, Yeongtong-gu, Suwon-si 16226, Republic of Korea; jsw0603@imdpharm.co.kr; 3 Bumin Hospital, 389, Gonghang-daero, Gangseo-gu, Seoul 07590, Republic of Korea

**Keywords:** EPA and DHA, clinical trial, liquid crystalline nanoparticle, pharmacokinetics, safety, bioavailability

## Abstract

Eicosapentaenoic acid (EPA) and docosahexaenoic acid (DHA) are well known for their capacity to lower triglyceride levels, but the clinical effectiveness is hindered by limited bioavailability and patient adherence. To address this challenge, we introduce a novel liquid crystalline nanoparticle-based formulation, the innovative medicine and drug delivery (IMD)-Omega soft capsule (cap), designed to optimize the pharmacokinetics (PK) and safety of EPA and DHA. This randomized, open-label, crossover study engages a cohort of 24 healthy adult subjects, utilizing key PK parameters like C_max_, AUC, T_max_, t_½_, and Ke to conduct a comprehensive evaluation. The trial compares the performance of the IMD-Omega soft cap with the well-established Omacor^®^ soft cap. The IMD-Omega soft cap exhibited an impressive 110% increase in bioavailability for EPA and a remarkable 134% surge for DHA in comparison to the Omacor^®^ soft cap over a span of 72 h. The key success can be attributed to the innovative liquid crystalline nanoparticle design, bolstering the dissolution and permeability of these essential fatty acids. Intriguingly, intra-participant variability for AUC_0–72_ h and C_max_ were calculated at 45.04% and 34.26%, respectively. It is noteworthy that the parameters of T_max_ for EPA (≈6.00 h) and DHA (≈5.00 h), t_½_ for both EPA and DHA ≈ 30–40 h, and K_el_ around 0.18–0.22 h^−1^ for EPA and ≈0.008–0.02 h^−1^ for DHA, displayed comparability between the IMD-Omega and Omacor^®^ formulations. Encouragingly, the IMD-Omega soft cap showed excellent tolerability. The promise of optimized patient compliance and reduced dosages adds further weight to its potential significance.

## 1. Introduction

Hyperlipidemia, a common worldwide disorder, is a condition characterized by abnormally high levels of low-density lipoprotein cholesterol (LDL-C), very-low-density lipoprotein cholesterol (VLDL-C), and triglycerides in the blood [1]. These increased plasma lipid concentrations have a significant link with cardiovascular diseases, such as angina and myocardial infarction, which happen to be the leading causes of mortality almost all over the world necessitates effective management strategies [2]. While maintaining a healthy weight through a balanced diet and regular exercise is important in managing hyperlipidemia, medication plays a crucial role as the keystone of treatment. The primary goal of managing hyperlipidemia is to reduce the levels of LDL-C, which is recognized as a major risk factor for cardiovascular diseases [3].

In the age of modern medication, high-intensity statins are often recommended as a first-line treatment for hyperlipidemia in reducing LDL-C levels. These statins are potent cholesterol-lowering drugs that have demonstrated effectiveness in lowering LDL-C levels and reducing the risk of cardiovascular incidents [4]. However, it is essential to acknowledge that statins also come with potential risks and side effects. One of the most concerning side effects associated with statin therapy is the development of rhabdomyolysis, a rare but potentially fatal complication, where damaged muscle fibers release their contents into the bloodstream, leading to kidney damage and other life-threatening complications [5]. Medical professionals can enhance hyperlipidemia therapy and improve patient outcomes by establishing a balance between medication and patient safety.

One promising therapeutic approach for managing hyperlipidemia involves the use of eicosapentaenoic acid (EPA) and docosahexaenoic acid (DHA), which are both long-chain ω-3 polyunsaturated fatty acids [6]. Numerous studies have demonstrated the usefulness of these fatty acids in considerably lowering triglyceride levels, particularly in persons with endogenous hypertriglyceridemia. Their ability to effectively lower triglyceride levels by inhibiting liver triglyceride production and reducing very-low-density lipoprotein cholesterol production makes them valuable tools in the management of this global health concern [7]. The administration of the ethyl ester forms of EPA and DHA has demonstrated remarkable success in treating hypertriglyceridemia. These fatty acids significantly reduce the amount of triglycerides in the circulation by limiting the liver’s synthesis of triglycerides. As a result, the production of very-low-density lipoprotein cholesterol (VLDL-C), which is directly linked to triglyceride levels, is also reduced. This mechanism of action not only reduce hypertriglyceridemia but also contributes to the overall reduction of atherogenic lipoproteins in the blood, thereby mitigating the risk of cardiovascular diseases [8].

The therapeutic potential of EPA and DHA has received significant attention, leading to the approval of several formulations by the U.S. Food and Drug Administration (FDA) as prescription drugs [9]. These formulations may come in the form of ethyl esters or carboxylic acid, providing healthcare professionals with various alternatives to tailor the treatment to individual patient needs. One particularly noteworthy aspect of ω-3 fatty acids therapy is their favorable safety profile [10]. Notably, they do not lead to addiction or tolerance, which makes ω-3 fatty acids an attractive option for patients requiring long-term management of hyperlipidemia.

Despite the well-proven efficacy of ethyl ester formulations, ω-3 fatty acids face significant challenges related to their bioavailability and absorption. When consumed in a fasting state, their bioavailability is relatively low, primarily due to their high lipophilicity, which influences their ability to reach the unstirred water layers of the gastrointestinal tract for absorption [11]. This limitation underscores the significance of the dietary context in which ω-3 fatty acids are ingested and the importance of understanding their interactions with other nutrients.

A well-known commercial ω-3 ethyl ester product is the Omacor^®^ soft capsule (cap) (Omacor^®^), which is typically prescribed at doses ranging from 2 to 4 g once or twice daily. However, a major drawback of such high dosages is the need for large soft gel caps to accommodate the substantial amount of the active ingredient [12]. This aspect has presented a considerable challenge in terms of patient compliance. Swallowing such large caps may be difficult for some individuals, leading to potential non-adherence to the prescribed dosage regimen. This, in turn, can compromise the effectiveness of the treatment. Moreover, the administration of high doses of ω-3 fatty acids has been linked to certain adverse effects. A common complaint is the presence of a fishy aftertaste, which can be unpleasant and can discourage patients from continuing the treatment. Additionally, some individuals may experience gastrointestinal disorders, such as burping, attributed to the oxidative degradation of the fatty acids and the formation of oil droplets in the gastrointestinal tract. These adverse effects can reduce patient satisfaction and have an influence on overall treatment adherence [13].

To address these challenges and optimize the therapeutic potential of ω-3 fatty acids, ongoing research is actively looking for alternative delivery methods and formulations. Microencapsulation, emulsions, and nanoemulsions are some of the innovative approaches being investigated to enhance the stability and absorption of these essential fatty acids. These developments aim to improve patient acceptability and overall treatment results by increasing bioavailability, lowering the necessary dose, and decreasing adverse effects. In our previous study, we made significant progress in addressing the limitations of current commercial ω-3 acid ethyl ester products by developing a groundbreaking liquid crystalline nanoparticle-based formulation called the IMD-Omega soft capsule (IMD-Omega) [14]. This innovative formulation was specifically designed to enhance the dissolution and permeability of ω-3 fatty acids, EPA, and DHA, thereby overcoming the bioavailability challenges faced by existing conventional products. Our earlier findings revealed that the optimal innovative medicine and drug delivery (IMD)-Omega formulation demonstrated a remarkable 1.7-to 2.3-fold increase in the bioavailability of EPA and DHA compared to the commercialized Omacor^®^. This enhancement in bioavailability was achieved through the spontaneous formation of liquid crystalline nanoparticles. The enhanced bioavailability holds significant promise as it can potentially lead to a reduction in the required dosage of ω-3 fatty acids, making it more convenient for patients and potentially enhancing treatment adherence.

Given these encouraging results, our current study aims to explore deeper the pharmacokinetics (PK) and safety profiles of the IMD-Omega formulation of ω-3 fatty acids on a randomized, open-label, single-dose, crossover trial of IMD-Omega in healthy adults. We will conduct comprehensive evaluations and comparisons with the commercially available Omacor^®^. Moreover, a critical aspect of this investigation involves an in-depth safety assessment of IMD-Omega. Understanding the potential side effects and safety profile of the formulation is essential in ensuring its clinical viability and patient acceptance. By evaluating and comparing the safety profiles with existing prescription drugs, we aim to identify any potential advantages or drawbacks of the novel formulation concerning patient tolerability and overall safety. The implications of this study extend beyond just addressing the challenges of current ω-3 fatty acid therapies. If successful, IMD-Omega could represent a major breakthrough in hyperlipidemia treatment, potentially offering a more effective and better-tolerated therapeutic alternative.

## 2. Results

### 2.1. Participants Characteristics

Table 1 provides a summarized overview of the demographic characteristics of the participants enrolled in this study. The investigation centered on a cohort of healthy adults aged 19 years or older. The study started with an initial participant count of 50, but following rigorous application of inclusion and exclusion criteria, a final selection of 24 participants emerged, all of whom completed the study without any dropouts after the initial administration of the investigational medicinal product.

The average age of the participants was calculated at 26.9 ± 6.8 years, exhibiting a range spanning from 19 to 45 years (*p* = 0.383). Similarly, the mean height and weight were determined as 175.1 ± 5.2 cm (with a range of 165.2 to 181.3 cm, *p* = 0.0585) and 72.2 ± 11.4 kg (ranging from 53.5 to 98.4 kg, *p* = 0.859), respectively. The computed mean body mass index (BMI) was 23.5 ± 3.3 kg/m^2^, with a range of 18.1 to 29.9 kg/m^2^ (*p* = 0.697). Importantly, the entirety of the participant cohort shared a common Korean ethnicity. Throughout the screening process, all participants exhibited normal or clinically insignificant diagnostic test results, thereby confirming their baseline health status. No anomalies, concomitant medications, or baseline characteristics that could impact the study outcomes were reported for any participant. These demographic details underscore the meticulous selection process, emphasizing a well-defined and homogenous participant cohort that was closely aligned with the study’s objectives.

### 2.2. Pharmacokinetics Comparison of IMD-Omega vs. Omacor^®^

**Figure 1:**
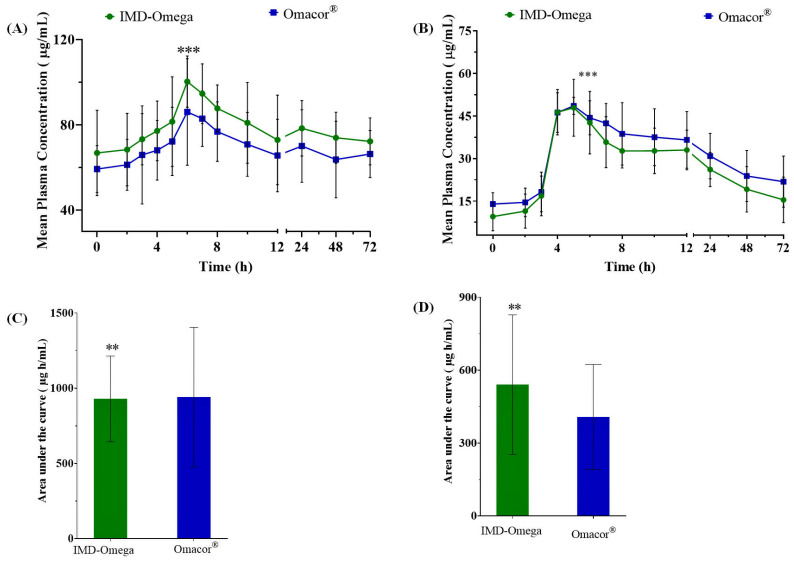
Pharmacokinetics of EPA and DHA in IMD-Omega and Omacor^®^. (**A**) Concentration (mg/dL) of plasma EPA in IMD-Omega and Omacor^®^ over 72 h. (**B**) Concentration (mg/dL) of plasma DHA in IMD-Omega and Omacor^®^ over 72 h. (**C**) AUC_0–72_ for the plasma EPA in IMD-Omega and Omacor^®^ over 72 h. (**D**) AUC_0–72_ or the plasma DHA in IMD-Omega and Omacor^®^ over 72 h. All results are expressed as mean ± SD (n = 12). *** *p* value < 0.001; ** *p* value < 0.005 (*t*-test).

The study thoroughly investigated the PK parameters and relative bioavailability of two distinct formulations, namely IMD-Omega and Omacor^®^, for both EPA and DHA. This evaluation was presented through Figure 1, which visually depicted the mean plasma concentration–time curve profiles, along AUC data, encompassing total EPA and total DHA concentrations over a 72 h interval following drug administration.

Notably, the C_max_ values for EPA and DHA were significantly higher for IMD-Omega in contrast to Omacor^®^ (EPA: 43.5 ± 17.3 μg/mL vs. 40.6 ± 23.7 μg/mL; DHA: 35.7 ± 16.6 μg/mL vs. 26.1 ± 16.4 μg/mL). The AUC_0–72_ h values for IMD-Omega similarly displayed a favorable increase relative to the commercialized Omacor^®^ (EPA: 929.8 ± 284.6 μg‧h/mL vs. 914.0 ± 463.6 μg‧h/mL; DHA: 540.5 ± 287.2 μg‧h/mL vs. 406.9 ± 216.1 μg‧h/mL). Importantly, the parameters of T_max_, t_1/2_, and K_el_ were found to be comparable for both formulations (Table 2). The specific T_max_ values for EPA were 6 h for both the test and reference samples, while for DHA, they were 5 h for the test and 6 h for the reference. These comprehensive insights into the PK profiles were further detailed in Table 2.

The t_1/2_ values for EPA and DHA within IMD-Omega were calculated as 30.9 ± 11.7 h and 34.8 ± 26.2 h, respectively. In contrast, Omacor^®^ exhibited t_1/2_ values of 40.1 ± 21.9 h for EPA and 84.1 ± 195.3 h for DHA.

The study discusses substantial differences in the [15] bioavailability and kinetics of EPA and DHA between the two formulations by carefully analyzing these pharmacokinetic parameters. These findings add to a deeper understanding of the IMD-Omega and Omacor soft caps’ pharmacokinetic efficiency and highlight the potential therapeutic benefits of IMD-Omega over the commercial product.

### 2.3. Bioavailability

The comparative evaluation between IMD-Omega vs. Omacor^®^ is briefly summarized in Table 3, which specifies the crucial AUC_0–72_ h and C_max_ ratios. Notably, for EPA, the geometric mean ratio of C_max_ stood at an impressive 122.98%, encompassed within a 90% CI ranging from 98.3% to 153.6%. A similar trend was observed for DHA, where the ratio reached a substantial 151.5%, characterized by a 90% CI that ranged from 123.1% to 186.6%.

The observed *p*-value of 0.0001 underscored the statistical significance of these findings. Furthermore, the geometric mean ratio of AUC_0–72_ for EPA was calculated to be 109.5%, accompanied by a 90% CI spanning from 92.4% to 129.8%. Similarly, for DHA, the AUC_0–72_ ratio was 134.3%, encapsulated within a 90% CI ranging from 108.1% to 166.8%. Once again, the associated *p*-value of 0.0001 highlighted the robust statistical significance of these outcomes.

### 2.4. Safety

The investigational medicinal product was given to a total of 24 people over the course of this trial, and it is worth noting that none of these participants had any treatment-emergent or significant adverse events. Furthermore, when the study drug was administered, careful vital signs and diagnostic examinations were performed. Importantly, none of these examinations indicated any clinically significant abnormalities or unusual results. It is also worth noting that no additional drugs were given during the research period, emphasizing the investigational product’s overall tolerability among this cohort of participants.

## 3. Discussion

Elevated levels of LDL-C [16] and triglyceride-rich lipoprotein are recognized risk factors for coronary heart disease, escalating the urgency of effective treatments. In cases of markedly high triglyceride levels, prescription ω-3 acid ethyl esters have demonstrated their ability to notably decrease triglyceride levels [17]. These compounds can be administered alongside statins to further optimize non-high-density lipoprotein cholesterol levels while concurrently lowering triglycerides. However, a vital factor in enhancing the efficiency of ω-3 acid ethyl esters is the need for a high-fat meal to induce adequate pancreatic lipase activity, ensuring maximum EPA and DHA absorption in the intestines [18].

An established player in this domain is Omacor^®^, now known as Lovaza^®^, which comes in the form of a 1 g cap containing 460 mg of EPA ethyl ester and 380 mg of DHA ethyl ester. Its approval by the FDA is directed towards addressing exceedingly high triglyceride levels (greater than 500 mg/dL or 5.6 mmol/L) [19]. Against this context, our research delves deeper into the world of ω-3 acid ethyl ester formulations, with an emphasis on IMD-Omega [20]. To improve absorption, this novel formulation combines the synergistic effects of phospholipids and oleic acids. Significantly, this invention requires a lower dose than Omacor^®^. Our hypothesis is that IMD-Omega has equivalent or maybe enhanced bioavailability when compared to marketed Omacor^®^.

The important feature of sample size determination was based on Omacor^®^ soft cap product licensing data. Drawing from a range of intra-subject variability for AUC_0–72_ and C_max_ for both EPA and DHA when administering 4 g of ω-3 fatty acid to healthy adults, which ranged from around 19.2% to 42.3%, and accounting for a significance level of 0.05 and an intra-subject variability of 30%, we chose a sample size of 24 participants (12 per group) to maintain an 80% probability of detecting a 20% difference between treatment groups, assuming a 20% dropout rate.

Our study revealed fascinating results. The 90% CI for C_max_ and AUC_0–72_, in particular, exceeded the acceptable bioequivalence range of 80% to 125%. This means that the test formulation of IMD-Omega differs significantly from the reference medication, the Omacor^®^ soft cap, in terms of bioequivalence. This trial finding shows that IMD-Omega has the ability to produce therapeutic blood levels of ω-3 acid ethyl esters at a lower dosage, less than 580 mg. This potential offers various benefits, including increased patient adherence, reduced pill load, and possibly improved cost-effectiveness.

These findings showed that IMD-Omega liquid crystal formulations successfully increased the bioavailability of ω-3 fatty acid ester through improved dissolution and permeation, resulting in the formation of a cubic structure that spontaneously formed nanoparticles of the smallest size in the gastrointestinal tract and improved the formulation’s solubility and dissolution. When this sort of self-assembled liquid crystal forms spontaneously, bile salts and lipolytic enzymes can further solubilize and degrade it, specifically in the small intestine. As a result, ω-3 fatty acid absorption was increased much more as compared to the commercialized cap Omacor^®^. An investigation of the metabolic fate of EPA and DHA revealed that DHA metabolism remained substantially unchanged following stomach oxidation, enhancing DHA bioavailability in comparison to EPA [4,21].

In terms of safety, a single dosage of ω-3 was well tolerated with no incidence of significant side effects. These findings are consistent with prior studies, as evidenced by the work of other researchers. Various delivery techniques have been investigated in the attempt to improve bioavailability. Microemulsions [22] and liquid crystalline nanoparticles, like Nature Made^®^ Omega-3 and AquaCelle^®^ Omega-3 products, have showcased the improved bioavailability of EPA and DHA. Nevertheless, our research ventures into unfamiliar territory by investigating a formulation that promotes the manufacture of liquid crystalline nanoparticles in situ for ω-3 administration. These findings give concrete proof that the IMD-Omega soft cap has the potential to have equivalent or even greater bioavailability, even at lower dosages than standard formulations. A marketed traditional cap of ω-3 is normally recommended at dosages of 2 to 4 g once or twice a day, whereas the IMD-Omega soft cap is a single dosage of 580 mg daily; it clearly indicates a reduction in daily dose and pill burden for patients compared to 1 g Omacor^®^. The potential benefits, spanning from improved patient compliance to simplified dosing regimens, highlight the importance of cutting-edge delivery techniques such as liquid crystalline nanoparticles in the pharmaceutical and nutraceutical industries.

In the context of a crossover study design, it is important to highlight that each participant effectively served as their own control. The newly developed method assisted in decreasing the impact of inter-participant variability, a characteristic that generally has a significant impact on the assessment of product bioequivalence.

The study approach was able to offer more precise and trustworthy insights into the comparative efficacy of the tested formulations by using this self-control mechanism. Furthermore, the incorporation of baseline correction into the analytical technique was critical in arriving at a more accurate evaluation of the actual drug availability resulting from the delivered therapeutic product. By removing the contribution of endogenous drug levels in plasma, the baseline correction approach effectively separated the effects of the medicinal product itself, improving the precision of the obtained results.

## 4. Materials and Methods

### 4.1. Formulation Clinical Trial Study

In this study, two different formulations of ω-3 acid ethyl ester products are analyzed: the novel IMD-Omega soft cap (ω-3 acid ethyl ester 90, 580 mg) manufactured by IMD pharm Inc., Suwon, Republic of Korea, and the commercially available Omacor^®^ soft cap (ω-3 acid ethyl ester 90, 1000 mg) produced by Kuhnil Pharmaceutical Co., Ltd., Seoul, Republic of Korea. The IMD-Omega soft cap represents the novel liquid crystalline nanoparticle-based formulation, which has shown promising results in enhancing the dissolution and permeability of ω-3 fatty acids. On the other hand, the Omacor^®^ soft cap serves as the reference drug, as it is one of the established commercially available formulations used in clinical practice.

### 4.2. Inclusion and Exclusion Criteria of the Participants

This study was carried out at the clinical research center of Bumin Hospital in Seoul, Republic of Korea. We recruited 50 healthy male volunteers aged 19 and above. Each participant underwent a thorough medical history review, a physical examination, and a blood test before being included in the study. To be eligible, participants needed to have a body mass index (BMI) within the range of 18 to 30 kg/m^2^.

Inclusion criteria required that participants did not have any significant congenital or chronic diseases requiring treatment within the past 5 years, and they should not have exhibited any pathological symptoms during the examination. Participants were also advised to avoid participating in any other clinical research utilizing investigational medications for at least 30 days prior to enrolling in this study.

Several exclusion criteria were set in place to ensure the integrity and reliability of the study conclusions. Individuals were excluded if they had recently consumed drug-metabolizing enzymes, such as barbiturates, within 30 days before the first administration of investigational drugs. Those with a history of fish oil allergies or allergic reactions to lipids were also excluded from the study. Furthermore, anyone taking medications that could potentially interfere with the study had to refrain from participation within 10 days before receiving the investigational drug. Participants who had been part of another clinical trial within 6 months prior to the first administration of the investigational drug were also ineligible. Blood donation within 2 months or component blood donation within 2 weeks before the first administration of the investigational product were additional exclusion criteria.

Individuals with certain medical conditions were not allowed to participate, including those with neurological, psychiatric, cardiovascular, pulmonary, endocrine, hematological, or immunological problems. Participants with GI tract, liver, or kidney diseases that might affect drug absorption, as well as individuals with excessive alcohol consumption (males > 14 drinks/week) within 1 month before the study, were also excluded. Smokers were not eligible to participate in the trial. Individuals with hypothyroidism, moderate-to-severe lipidemia (total cholesterol ≤ 240 mg/dL, LDL ≤ 160 mg/dL, and TG ≤ 199 mg/dL), systolic blood pressure over 160 mmHg, diastolic blood pressure higher than 95 mmHg, or resting cardiac output less than 40 beats/min or over one hundred beats/min were excluded from the study.

**Figure 2:**
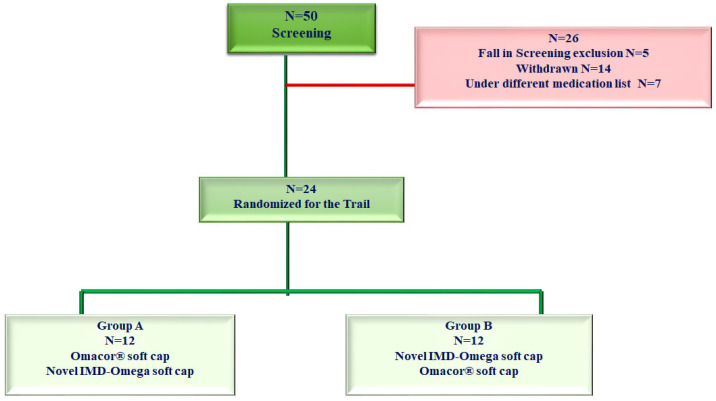
The inclusion and exclusion criteria for the clinical trial.

Eligibility for participation was determined by the study investigator based on each participant’s medical history and the results of the screening tests. Before agreeing to participate, all subjects received a comprehensive explanation of the study’s purpose, design, and procedures (Figure 2). They provided written informed consent, signifying their understanding and voluntary willingness to participate.

### 4.3. Objectives and Assessments of the Clinical Trial

The principal aim of this study revolved around a crucial comparison—the assessment of the AUC–time curve values—between the commercialized Omacor^®^ and our novel IMD-Omega formulation, both of which were administered under inclusion/exclusion criteria. The goal of these clinical trials was to offer the full knowledge of how each formulation’s concentration profile changed over time and how it correlated with overall exposure to the medicine over a specified time frame. Additionally, the study encompassed several secondary objectives that were of paramount importance. One such objective was the meticulous monitoring of the safety and tolerability of both formulations. By closely observing any adverse effects or potential discomfort experienced by the participants, the study aimed to ensure that the administration of the formulations was well-tolerated and posed minimal risk to the subjects. Furthermore, another secondary objective involved a comparison of three key parameters: C_max_, T_maxa_, and t_1/2_. These data gave critical insights into the formulations’ rate of absorption and the peak concentration levels obtained in the participants’ circulation following a single oral dosage administration.

### 4.4. Study Design

This study utilized a randomized, open-label, single-dose, crossover design to assess the PK of two different ω-3 acid ethyl ester formulations. The randomization process was conducted by a biostatistics expert, who assigned each participant a unique random number in chronological order of enrollment. This number determined the sequence order of treatment administration for each participant, either starting with the novel IMD-Omega and then moving on to Omacor^®^ or vice versa.

Importantly, no participants were excluded from the study after randomization, ensuring the integrity of the randomized design. Each participant received a single dose of either the test drug (IMD-Omega) or the reference drug (Omacor^®^), orally administered as four capsules, along with 200 mL of water. As an open-label trial, participants were aware of the formulation they were receiving for the efficacy study.

**Figure 3:**
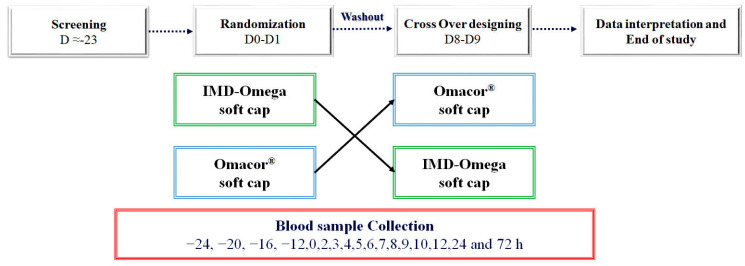
Randomized, crossover clinical trial study design: Primary screening started before the 23 days of the last day of the trial. The first randomization was performed on days 0 and 1, when participants tested the test and the reference sample. Seven days was the washout period of the study, and then the crossover was continued on days 8 and 9. For each sample, a blood sample was collected.

Blood samples were collected at various time points. The first blood sample was obtained after the participants had fasted for −24, −20, −16, −12, and 0 h before intake of the supplements. Subsequent blood samples were collected at 2, 3, 4, 5, 6, 7, 8, 9, 10, 12, 24, and 72 h (Figure 3) after the single dose intake, resulting in a total of 18 blood samples drawn for analysis.

To minimize the influence of meals on the pharmacokinetics of the supplements, participants were instructed to consume a standardized breakfast consisting of 600–750 kcal with a fat content of 15–20% within 20 min before medication administration.

Additionally, various screening tests were conducted on the participants, including hematology, blood chemistry, urine, and serology tests. Specifically, blood chemistry tests for AST (GOT), ALT (GPT), and γ-GTP were performed. Post-administration blood chemistry tests were conducted 72 h after each drug administration to assess potential changes in liver function.

Blood samples were processed via centrifugation, separating the plasma from white and red blood cells, and stored at −80 °C for further analysis. The study design is summarized in Table 4.

### 4.5. Blood Sample Collection and Analytical Method Development

Blood sampling was meticulously conducted across a total of 18 distinct time points for each participant, encompassing both pre-dose and post-dose periods. These samples were obtained via a heparin-locked catheter inserted into the participant’s vein, ensuring a controlled and consistent collection process. Throughout the trial, a cumulative volume of 280 mL of blood was meticulously gathered for analysis. The collected blood was subjected to centrifugation at 3000 rpm and 4 °C for a duration of 10 min to isolate the plasma. This plasma was then carefully stored in a refrigerator at −80 °C until transfer to a specialized clinical trial laboratory, DT&CRO in Yongin, Republic of Korea, where further analysis was conducted.

To prepare the plasma samples for analysis, a systematic protocol was followed. Initially, 50 mL of plasma was combined with 10 µL of DHA-d5 (100 g/mL), 50 µL of 10% Tween 80, and 500 µL of acetonitrile within a glass tube. The mixture underwent vortexing at 2500 rpm for a duration of 3 min. Subsequently, it was treated with a 500 µL combination of hydrochloric acid and acetonitrile (1:4, *v*/*v*), mixed again, allowed to sit for 60 min, and then cooled. This mixture was further treated with 1000 µL of hexanes and subjected to vortexing. Following centrifugation at 4000 rpm for 15 min, the organic layer’s upper portion, totaling 150 µL, was dried using nitrogen gas at a temperature of 40 °C. The resulting residue was then reconstituted with 1000 µL of 2 mM ammonium acetate, subjected to another round of mixing, and centrifuged at 13,500 rpm for 5 min. The upper layer of this process was transferred to a vial and mixed with an additional 100 µL of 2 mM ammonium acetate before being vortexed. The final mixture was then injected into a liquid chromatography–tandem mass spectrometry (LC-MS/MS) system for analysis.

The measurement of the total plasma concentration of EPA and DHA involved the use of sophisticated equipment. An ultra-fast liquid chromatography system from AB SCIEX in Los Angeles, USA, was employed for this purpose. This system featured a C18 reversed-phase column (3 µm, 2.1 mm × 50 mm; Waters, Milford, CT, USA) connected to a TQ 5500 tandem mass spectrometry system (AB SCIEX, Los Angeles, CA, USA) equipped with an electrospray ionization source. The mobile phase consisted of a mixture of 2 mM ammonium acetate (0.1% formic acid) and acetonitrile (15:9, *v*/*v*). During the analysis, the injection volume was set at 1 µL, and the flow rate was adjusted to 35 µL/s. The column oven temperature was maintained at 10 °C to ensure accurate measurements. The operation of the mass spectrometry system was effectively controlled using Analyst software (version 1.7.0, AB SCIEX, Los Angeles, CA, USA).

### 4.6. Pharmacokinetic Evaluation and Statistical Analysis

The PK parameters examined during the clinical trial were C_max_, AUC from 0 to 72 h (AUC_0–72_), AUC∞, T_max_, K_el_, and t_½_. PK parameters were calculated using noncompartmental methods with SAS^®^ Version 9.4 (SAS Institute Inc., Cary, NC, USA). Concentration values falling below the limit of quantification (BLQ) were considered as zero for PK parameter estimation. Baseline corrected data were employed for PK result analysis.

To account for the endogeneity of EPA and DHA, the baseline was adjusted for each component. C_max_ and AUC_t_ were analyzed for statistical significance by comparing the mean values using a 90% confidence interval (CI) after logarithmic transformation. To provide a comprehensive overview of the PK parameters at each sampling time, descriptive statistics were computed. For inferential statistical analyses, Statistical Analysis System (SAS) version 9.2 was utilized [8]. Analysis of variance (ANOVA) was applied to log-transformed AUC_0–72_ h and C_max_, while untransformed T_max_, K_el_, and t_½_ of EPA and DHA were subjected to PROC general linear model procedures. For a comprehensive understanding of variability, both intra- and inter-participant coefficient of variation (CV%) were calculated. To ensure accuracy, a drug potency correction was executed, given that the measured drug content of the test formulation deviated more than 5% from that of the capsule formulation.

### 4.7. Safety Assessment of the Formulation

Throughout the trials, participants’ safety was thoroughly evaluated using a comprehensive examination that covered every aspect of their well-being. These measurements were conducted at the 24 h, 48 h, and 72 h marks post drug administration. This assessment involved the vigilant monitoring of adverse events, along with the meticulous observation of vital signs such as blood pressure, heart rate, and body temperature. In addition, specific blood chemistry parameters were closely examined, including alanine aminotransferase, gamma-alanine aminotransferase, and aspartate aminotransferase. Furthermore, blood chemistry tests were thoughtfully executed on the third day of the trial, providing valuable insights into specific enzymatic activities associated with liver function. These tests specifically focused on alanine aminotransferase, gamma-alanine aminotransferase, and aspartate aminotransferase levels, thereby offering a comprehensive overview of potential metabolic alterations. By implementing this meticulous safety assessment approach, the study aimed to promptly identify and address any adverse effects, ensuring the well-being and health of the participants throughout the trial period. This commitment to safety underscores the ethical and responsible conduct of the research, prioritizing the participants’ health and ensuring the reliability of the study’s findings.

### 4.8. Ethics of the Clinical Trial

The entire clinical study was meticulously conducted in strict adherence to the highest standards of ethical and scientific conduct. The study’s execution followed the principles of Good Clinical Practices (GCP) and Good Laboratory Practices (GLP), both of which are firmly established frameworks for ensuring the integrity and reliability of clinical research. These practices were aligned with the guidelines set forth by the International Council for Harmonization of Technical Requirements for Pharmaceuticals for Human Use (ICH), particularly ICH E6 [R2]. Local regulatory standards were strictly respected, as were the most recent versions of the World Medical Association and the Declaration of Helsinki. These guidelines collectively ensure the protection of participants’ rights, well-being, and data integrity throughout the study. The study underwent a rigorous ethical review and was granted approval by the Institutional Review Board (IRB) of Seoul Bumin Hospital (IRB number: BE_20_033, Seoul, Republic of Korea). Furthermore, before engaging in any study-related procedures, all participants were provided with a thorough explanation of the study’s purpose, design, procedures, and potential risks and benefits. This transparent disclosure allowed participants to make informed decisions about their involvement. To formally indicate their informed consent and willingness to participate, each participant signed a written informed consent document.

## 5. Conclusions

In conclusion, this study has provided a comprehensive assessment of the PK attributes and safety aspects associated with the IMD-Omega soft cap formulation based on liquid crystalline nanoparticles of EPA and DHA. Through a rigorous evaluation and comparison with the established Omacor^®^ soft cap, our findings have revealed the remarkable potential of IMD-Omega in terms of the enhanced bioavailability of EPA and DHA compared to the marketed formulations. The formulation demonstrated a notable 110% improvement in EPA bioavailability and a remarkable 134% increase in DHA bioavailability, which was attributable mostly to the unique liquid crystalline nanoparticle design, which facilitated increased ω-3 fatty acid solubility and permeability. Safety considerations remained paramount throughout this investigation, and study observations underscore the excellent tolerability of the IMD-Omega soft cap. With no instances of treatment-emergent or serious adverse events recorded among the study participants, data confirm the formulation’s robust safety profile for healthy adults. These encouraging outcomes support the IMD-Omega soft cap as a promising therapeutic avenue for addressing hyperlipidemia. Notably, its potential to effectively reduce required dosing and enhance patient compliance by virtue of its improved tolerability is a noteworthy stride towards optimizing treatment regimens. The potential of reaching equivalent bioequivalence to the Omacor^®^ soft cap at a reduced dosage of about 500 mg enhances our confidence in IMD-Omega’s transformative opportunities. The potential demonstrated by IMD-Omega necessitates further research into the therapeutic importance and broader positive effects of this formulation on triglyceride lowering and hyperlipidemia. The potential of modifying hyperlipidemia treatment strategies remains tempting as time goes by.

## Figures and Tables

**Table 1:** Participants characteristics: age, sex, height, weight, and BMI.

Characteristics	A (*n* = 12)	B (*n* = 12)	Total (*n* = 24)
Age (y)	26.3 ± 8.1 (19–45)	27.6 ± 5.7 (19–37)	26.9 ± 6.8 (19–45)
Sex, male, n (%)	12 (100.0)	12 (100.0)	24 (100.0)
Height (cm)	175.7 ± 5.1 (165.7–181.2)	174.5 ± 5.41 (165.2–181.3)	175.1 ± 5.2 (165.2–181.3)
Weight (kg)	71.8 ± 10.3 (56.5–88.1)	72.7 ± 12.8 (53.5–98.4)	72.2 ± 11.4 (53.5–98.4)
Body mass index (kg/m^2^)	23.2 ± 3.1 (18.3–28.1)	23.8 ± 3.6 (18.1–29.9)	23.5 ± 3.3 (18.1–29.9)

A: Group with test drug followed by reference drug in sequence. B: Group with reference drug followed by test drug in sequence.

**Table 2:** Summary of the pharmacokinetic characteristics of EPA and DHA in the test and reference drugs (baseline corrected for each treatment).

Pharmacokinetic Parameters	IMD-Omega (Teste Sample)	Omacor^®^ (Reference Sample)
**Total EPA pharmacokinetics**		
C_max_ ^†^ (μg/mL)	43.5 ± 17.3	40.6 ± 23.8
AUC_t_^†^ (μg‧h/mL)	929.8 ± 284.6	914.0 ± 463.6
AUC_∞_^†^ (μg‧h/mL)	1216.8 ± 437.8	1412.6 ± 849.5
T_max_^‡^ (h)	6.0 (3.0–12.0)	6.0 (5.0–12.0)
t_1/2_^†^ (h)	30.9 ±11.7	40.1 ±21.9
K_el_ (h^−1^)	0.02 ± 0.01	0.02 ± 0.01
**Total DHA pharmacokinetics**		
C_max_ ^†^ (μg/mL)	35.7 ± 16.6	26.1 ± 16.4
AUC_t_^†^ (μg‧h/mL)	540.5 ± 287.2	406.9 ± 216.2
AUC_∞_^†^ (μg‧h/mL)	867.8 ± 834.2	1899.4 ± 4993.1
T_max_^‡^ (h)	5.0 (3.0–72.0)	5.0 (3.0–72.0)
t_1/2_^†^ (h)	34.8 ± 26.2	34.8 ± 195.3
K_el_ (h^−1^)	0.02 ± 0.01	0.01 ± 0.01

^†^ Arithmetic mean. ± Standard deviation. ^‡^ Median (range).

**Table 3:** Treatment comparison ratios for of EPA and DHA in test and reference drugs using a corrected baseline.

Parameter	Geometric LS-GMR		Average	Ratio (%)	90% CI	Inter-Patient CV (%)	Intra-Patient CV (%)
	Test Drug N = 12	Reference Drug N = 12					
**Total EPA**							
AUC_t_ (μg‧h/mL)	876.1	799.9	1.1	109.5	0.9 ≤ δ ≤ 1.3	14.7	34.2
C_max_ (μg/mL)	40.3	32.7	1.2	122.9	0.9 ≤ δ ≤ 1.5	12.5	45.1
**Total DHA**							
AUC_t_ (μg‧h/mL)	347.1	466.2	1.3	134.3	1.1 ≤ δ ≤ 1.7	7.3	43.7
C_max_ (μg/mL)	21.0	31.9	1.5	151.5	1.3 ≤ δ ≤ 1.9	12.9	41.9

LS-GMR: least-squares geometric mean ratio; CV: coefficient of variation.

**Table 4:** Two group administration plans by cross-over design.

Order Group	Number of Test Subjects	1st Period	Drug Wash out Period	2nd Period
A	12 people	Test drug	7 days	Reference drug
B	12 people	Reference drug		Test drug

## Data Availability

Data is contained within the article.
